# Study protocol for the SECURE (RAFT‐5) study: Service Evaluation of in‐hospital Cross‐speciality UK adult Rapid sequence intubation Events

**DOI:** 10.1002/anr3.70071

**Published:** 2026-07-08

**Authors:** T.E. Baumer, S. Gupta, T.W. Davies, S. Tomassini, S.R. Harrogate, A.V. Ramesh, T.E. Cloke, C. Taylor, C. Lim, J.D. Barnes, I. Grossi, S. Sudan, T.M. Cook, SECURE (RAFT‐5) Investigation Group (STAR, RAFT, TRIC, TERN and EMAR)

**Affiliations:** ^1^ Department of Anaesthesia and Intensive Care Royal United Hospitals Bath NHS Trust UK; ^2^ Department of Anaesthesia Gloucestershire NHS Foundation Trust Gloucester UK; ^3^ Faculty of Medicine & Dentistry Queen Mary University of London London UK; ^4^ Clinical Trials Unit Warwick Medical School Coventry UK; ^5^ Department of Anaesthesia North Bristol NHS Trust Bristol UK; ^6^ Department of Intensive Care North Bristol NHS Trust Bristol UK; ^7^ Bristol Heart Institute University Hospitals Bristol and Weston NHS Foundation Trust Bristol UK; ^8^ Emergency Department University Hospitals Bristol and Weston NHS Foundation Trust Bristol UK; ^9^ Department of Anaesthesia University Hospital Sussex NHS Foundation Trust Brighton UK; ^10^ SECURE (RAFT‐5) Investigation Group, UK (Severn Trainee Anaesthetic Research group; Research and Audit Federation of Anaesthetists in Training; Trainee Research in Intensive Care; Trainee Emergency Research Network; and Emergency Medicine Airway Registry)

**Keywords:** adults, in‐hospital, rapid sequence intubation

## Abstract

Rapid sequence intubation has evolved substantially since its inception over 50 years ago. Contemporary in‐hospital rapid sequence intubation practice includes a combination of variably employed components, many of which lack robust supporting evidence. Real‐world practice and the beliefs underpinning it are poorly characterised. We present the protocol for SECURE (Service Evaluation of Cross‐speciality United Kingdom Rapid Sequence Intubation Events), a prospective service evaluation which aims to characterise adult in‐hospital rapid sequence intubation practice across the United Kingdom. SECURE will comprise three electronic surveys (with data collection windows): (1) a Site Survey (six weeks) assessing local resources and governance; (2) a Vignette‐based Survey (seven weeks) exploring drivers of variation in self‐reported rapid sequence intubation practice; and (3) an Activity Survey (14 days per site) capturing user‐defined rapid sequence intubation events and immediate complications. Survey development followed the Consensus‐Based Checklist for Reporting of Survey Studies and was reviewed by expert advisers. Data will be stored securely at the Sponsor site and analysed in collaboration with a statistician. SECURE will provide a national overview of in‐hospital rapid sequence intubation practice, including variation in resources and decision‐making and describe the incidence of immediate complications. Formally recognised as a service evaluation, the study will be registered with information governance departments and represents the fifth project of the Research and Audit Federation of Anaesthetists in Training, overseen by the SECURE Investigation Group. Findings will inform future recommendations, investment and research in rapid sequence intubation practice.

## Introduction

Rapid sequence intubation (RSI) was described in 1970 as a prescriptive 15‐step process to reduce the risk of pulmonary aspiration after induction of anaesthesia and before securing a cuffed tracheal tube [1]. Over 50 years, the technique has changed with advances in equipment and pharmacological agents. Examples include the introduction of videolaryngoscopy, high flow nasal oxygenation, substitution of thiopentone for other induction agents, the use of rocuronium for neuromuscular blockade and the advent of sugammadex for its rapid reversal [2, 3, 4, 5, 6, 7]. The term RSI, or ‘modified’ RSI, now refers to a variety of components employed in various combinations during a single episode of airway management.

Lessons learnt from the fourth National Audit Project (NAP4) of the Royal College of Anaesthetists (RCoA) and the Difficult Airway Society (DAS) improved the identification of patients at risk of pulmonary aspiration and the appropriate adoption of RSI strategies [8]. Aspiration still accounts for 10% of airway and respiratory‐related cardiac arrests in the peri‐operative period [9]. Large studies have found that RSIs are associated with complications, such as difficult intubation, hypoxaemia and cardiovascular instability [8, 10, 11, 12, 13]. Such associations may be due to patient characteristics and the clinical urgency of airway management [11] but the execution or omission of various components of the RSI strategy may play a role. Recent deaths associated with RSI have led to coronial reports for the prevention of future deaths and have highlighted clinical variation in beliefs and practices [14, 15].

Several guidelines with reference to RSI have been published by the DAS in the UK: ‘management of difficult and failed tracheal intubation in obstetrics’; ‘tracheal intubation in critically ill patients’; and most recently in 2025, ‘management of unanticipated difficult tracheal intubation’ [16, 17, 18]. The RCoA, the Association of Anaesthetists (AoA) and DAS recently acknowledged that key elements of RSI lack robust supporting evidence and emphasised the urgent need for best practice statements based on contemporary data to ensure patient safety [14, 19, 20]. Many elements of RSI practice remain topics of debate and controversy, including when RSI is indicated, use of a nasogastric tube to empty stomach contents, drug choices and particularly the value of applying cricoid force. Views surrounding RSI may vary significantly across anaesthesia, intensive care medicine (ICM) and emergency medicine (EM).

A broad overview of contemporary UK practice can be gained by drawing from multiple interval surveys on RSI performance – these show significant variation in practice, although there may be a discrepancy between reported and actual clinical behaviour [4, 21]. Notably, there has been no UK‐wide registry study examining RSI practice across all specialities which employ the technique. We hypothesise that there will be wide variation in resources, actual team practice and individual self‐reported practice in relation to RSI events across different clinical areas, hospitals and regions in the UK.

SECURE will aim to characterise the delivery of in‐hospital RSI in adult patients by cross‐speciality teams in UK National Health System (NHS) hospitals. The study objectives will be to: (1) describe the availability of resources surrounding RSI events; (2) evaluate drivers of variation in individual self‐reported RSI practice across vignettes; (3) capture a snapshot of RSI events to describe contemporaneous practice; (4) describe the incidence of immediate complications of RSI; and (5) compare the findings to recent or upcoming national and international RSI guidance, including DAS, RCoA and Project for Universal Management of Airways (PUMA) [14, 22].

## Methods

### Patient and public involvement

Contemporary RSI practice was judged to be an important topic by patients and clinicians when chosen in a competitive tender of projects by the Research and Audit Federation of Anaesthetists in Training (RAFT). The RCoA Patient, Carer and Public Involvement and Engagement (PCPIE) Group has reviewed all elements of SECURE and provided strong support. The study has endorsement from the DAS, the Society for Intravenous Anaesthesia (SIVA), the RCoA and the Royal College of Emergency Medicine (RCEM).

### Study participation

SECURE will be a cross‐sectional, prospective service evaluation, consisting of three separate surveys relating to RSI events. All NHS hospitals in the UK where RSI is performed, with clinical services provided by anaesthesia, ICM or EM will be eligible for participation. Each individual NHS hospital will be considered a study site, and we will target enrolment of over 200 sites. Non‐NHS hospitals will not be eligible for logistical reasons.

SECURE is the fifth official RAFT project (RAFT‐5) in collaboration with Severn Trainee Anaesthetic Research (STAR), Trainee Research in Intensive Care (TRIC), Trainee Emergency Research Network (TERN) and Emergency Medicine Airway Registry (EMAR) groups. SECURE will be overseen by our national Investigation Group, which includes an expert advisory panel of consultants. The project will be led and delivered by resident doctors, with all residents (or appropriately qualified allied health professionals) in anaesthesia, ICM and EM able to participate at eligible, registered study sites.

The Investigation Group has established a SECURE network of regional leads to cover the three specialities across every UK training region. Regional leads will help recruit and support investigator teams at each study site that registers in their region. Investigator teams will include site leads, who will in turn recruit cross‐speciality consultant advocates and local investigators for support. Investigator teams will facilitate data collection by practitioners involved in team RSI events.

### Survey design

#### Site survey

The Site Survey will be an evaluation of baseline resources for supporting RSI events. The survey will capture typical provision in the single highest‐acuity location, where the majority of RSIs are performed in four key clinical areas: operating theatres (excluding obstetric theatres), obstetric theatres, intensive care units and emergency departments (ED). Depending on the clinical services available, the survey will be completed up to a maximum of four times per site.

The survey case report form will cover four domains: hospital characteristics, physical equipment, staff infrastructure and information governance. The domains will focus on equipment availability (and where relevant, timeframes of availability), including monitoring, oxygenation, suctioning, difficult access, laryngoscopy, tracheal intubation, difficult airway management and ventilation. Additionally, drug accessibility, personnel, staff training, local policy and learning from incidents will be included. The results of the survey will help inform how RSI preparedness varies across the UK using measures derived from DAS and PUMA guidelines (see Supporting Information, Appendix S1) [16, 17, 18, 22].

#### Vignette‐based survey

The Vignette‐based Survey will evaluate respondents' selection of optional airway management components across a series of clinical vignettes. Eligible participants will be clinical practitioners in anaesthesia, ICM or EM who are airway‐trained (defined as having completed or reached the equivalent level of competence as that described in the RCoA Initial Assessment of Competency) and involved in RSI decision‐making [23]. The case report form will include key characteristics, such as age, sex, ethnicity, training background, job role, seniority grade and intubation or RSI experience.

A ‘baseline vignette’ (without any specific driver of variation in practice) will act as a comparator [24, 25]. This will be followed by around six ‘experimental vignettes’, each with a single potential driver of variation in practice in a randomised order. These may include scenarios, such as increased risk of pulmonary aspiration and physiologically difficult airways [26]. For each vignette, selected identical airway management components will be presented to each respondent for comparison with their baseline.

Finally, practitioners with an anaesthetic training background will be presented with seven short vignettes and quick‐fire questions on aspiration risk specifically looking at drug administration technique, nasogastric tube insertion and use of cricoid force in more detail. The inclusion of vignettes/components in the survey will be decided using existing guidelines and Delphi studies, alongside expert advisor opinion and feedback from survey piloting [16, 17, 18, 22, 26]. The exact selection will not be openly publicised in advance to avoid biasing practitioners' responses.

#### Activity Survey

The Activity Survey will provide a snapshot of actual RSI events by intubating teams. Rapid sequence intubation events will be user‐defined (any intubation the reporter considers to be an RSI), with specific patient selection criteria (Table 1). We intend to capture any heterogeneity under the umbrella terms RSI or modified RSI; teams will self‐identify intubation events where RSI‐defining features are perceived to be present. The user‐defined approach is designed to encompass the full range of real‐world RSI behaviour and will avoid the risk of misclassification or bias associated with a formal, external definition.

**Table 1:** Activity Survey definitions and patient selection criteria.

Study definitions	**User‐defined RSI** – any intubation deemed to be an RSI (or modified RSI) by the intubating team **Immediate complications** – complications occurring in the period between the start of induction of anaesthesia and up to 10 min after the airway has been secured
Inclusion criteria	User‐defined RSI events that meet all the following criteria: In‐hospital (within study site grounds) Patient ≥ 18 years (known or believed) By anaesthetic, ICM and/or EM teams *Repeat RSIs in the same patient will be included as a new event*
Exclusion criteria	User‐defined RSI events meeting any of the following criteria: Out‐of‐hospital (outside of study site grounds) Patient < 18 years of age (known or believed) Not performed by anaesthetics, ICM or EM teams Patient with a functioning tracheostomy in‐situ Intubation during cardiac arrest (unless after ROSC)

RSI, rapid sequence intubation; ICM, intensive care medicine; EM, emergency medicine; ROSC, return of spontaneous circulation.

Intubating teams at every site will be encouraged to complete a case report form for all user‐defined RSI as soon as possible after the event. The case report form will evaluate situation factors, patient characteristics and procedural aspects. We will capture pre‐specified immediate complications in the period between induction of anaesthesia and up to 10 min after the airway has been secured (Table 2). Core outcomes from Airway Terminology and Outcome Measures (ATOM) will be followed as closely as possible, where available, for airway‐specific immediate complications [27].

**Table 2:** Activity Survey CRF summary.

Section	Question domains
Situation factors	Study site Timing (in hours/out of hours) Hospital area
Patient factors	Age Sex Ethnicity Height/weight (or body mass index) American Society of Anaesthesiologists (ASA) grade Clinical frailty score Indication for RSI Urgency of intubation Risk factors for pulmonary aspiration Predicted /known airway difficulty
Technical factors	RSI checklist verbalisation and allocation of team roles Prokinetic/antacid administration Nasogastric tube management Working suction availability Bed movement/positioning Pre‐oxygenation methods Cardiovascular status and monitoring Drug selection/administration Airway management including: Apnoeic oxygenation/ventilation Laryngoscope blade/adjuncts Cricoid force decision‐making and practice Intubation (including success or rescue strategies)
Immediate complications	**Airway** – trauma, tracheal tube misplacement, laryngospasm, difficult airway (single and composite outcomes) **Respiratory** – hypoxaemia, pulmonary aspiration (gastric contents or blood), bronchospasm, pneumothorax **Cardiovascular** – hypotension, hypertension, arrhythmias, cardiac ischaemia, cardiac arrest, death

### Primary outcomes

Given SECURE is a service evaluation with a range of study objectives, there will not be an overall pre‐specified primary outcome for the entire study – each survey will have its own defined primary outcome, as follows:

1. Site Survey – proportion of clinical areas meeting an essential RSI resource standard (RSI preparedness);
2. Vignette‐based Survey – the proportion of respondents with any change from baseline in their selection of RSI components across clinical vignettes;
3. Activity Survey – proportion of RSI events with first attempt success at tracheal intubation and without immediate complications related to airway management.

The Site Survey was completed by investigator teams at all registered study sites during a 6‐week data collection window (01/03/2026 to 12/04/2026). The Vignette‐based survey will be open for practitioner responses over a seven week period between 13/07/2026 and 30/08/2026. During the Activity Survey, prospective data was collected by intubating teams over a continuous 14‐day period within a broader data collection window (13/04/2026 to 25/05/2026). Exact dates were agreed at each study site to enable simultaneous data collection across all clinical areas locally. We will aim to complete full data analysis and reporting sequentially for all three surveys by mid‐2027.

The study surveys will be publicised and delivered widely through the SECURE investigator network at a local level. We will target an 80% response rate for the Site Survey per clinical area (> 500 responses in total). The Vignette‐based Survey will be distributed across all sites, with a target of at least 5% of eligible practitioners (> 1300 responses). A final response rate will be calculated using data from NHS bodies and colleges – approximately 27,000 practitioners work across anaesthesia, ICM and EM. We assume all will be eligible apart from a small number of residents who have not achieved their competency in RSI (Supporting Information, Appendix S2).

We estimate around 7400 in‐hospital adult RSI events (approximately 25% out‐of‐theatre) occur UK‐wide in a typical 14‐day period. This number is extrapolated from previous UK‐based studies, including large snapshot surveys, which suggest RSI rates are reproducible by time and place (Supporting Information, Appendix S3) [9]. The denominator for the survey will be scaled to registered sites – targeting > 10% capture (> 740 RSI events, assuming all eligible sites participate).

We will try to reduce selection bias in all three surveys by promoting SECURE widely across our cross‐speciality network. The surveys will have cross‐speciality usability due to close collaboration during the design stage. To assess for site‐level selection bias in the Activity Survey, we will report the representation of sites and RSI events by hospital size and type and clinical services. Selection bias at the clinician level has been considered, and the case report form has been streamlined as much as possible to facilitate entry of complex cases. Team recall bias will be mitigated by contemporaneous case report form completion, where possible and the presence of ‘unknown’ options to avoid inaccurate responses.

The Vignette‐based Survey may result in bias relating to self‐selection. We will measure respondent characteristics and report the distribution of survey responses, for example, by region, speciality and experience. We will design and pilot questions with real‐world applicability, to minimise the artefact between self‐reported and actual practice (‘the say‐do‐gap’). Non‐RSI‐competent practitioners, who may be more prone to misinterpretation and the observer (Hawthorne) effect, are thus excluded. Vignette randomisation will be used to reduce priming effects potentially associated with a pre‐set order.

Survey questions have been informed by current literature, intubation registries, such as the Emergency Medicine Airway Registry (EMAR), and recommendations from our collaborators, including DAS review of a draft case report form for the Activity Survey. Cross‐speciality consultant expert adviser input was sought throughout the design process. Each survey follows an international consensus‐based checklist methodology for reporting of survey studies (CROSS) [28]. Internal piloting feedback from members of our Investigation Group across multiple sites was sought for each survey.

All case report forms will be built by our electronic survey design expert using REDCap (https://project‐redcap.org), a secure web application for online surveys and made available via posters with a QR code and a weblink. The surveys will contain pre‐populated categorical answers, including related branching questions and some pre‐defined numerical ranges. All questions will be single or multiple‐choice, with an option for ‘other’, where relevant.

### Data management

Data collected for all three surveys will be uploaded promptly to a central, secure REDCap database at the study Sponsor site. After submission, to ensure compliance with data protection and information governance standards, no patient‐identifiable information will be accessible by local teams. Database access will be restricted to selected members of the SECURE Investigation Group, who will monitor the data regularly for accuracy and completeness.

The study statistician will undertake data cleaning for each survey once data collection is complete. Fully completed case report forms will be included in the final analysis, while incomplete or duplicate submissions will be excluded. Where possible, local teams will be asked to report duplications and errors centrally by email to assist in identification by site, date and time stamp. Where there is any uncertainty around the inclusion of a case report form response in analysis, the investigation group will make a final decision on a case‐by‐case basis.

Our project statistician has reviewed the design of each survey case report form to ensure consistency with the planned statistical analysis. As a descriptive service evaluation, no formal sample size calculation was performed. After analysis, data will be archived in accordance with the information governance policy of the Sponsor site for a maximum of five years, with the Chief Investigator acting as the custodian.

Initially, a basic descriptive analysis will be undertaken for all three surveys, including absolute numbers and percentages within categorical groups and medians (IQR), where applicable. We will report our outcomes, summarise responses to survey questions, identify themes and consensus points and explore variability in responses. Of interest, we will report by sub‐groups cross‐speciality, between different types of NHS hospitals and regions in the UK.

An additional analysis plan for the Vignette‐based Survey and Activity Survey will be determined in advance. All tests will be two‐tailed, and where possible, odds ratios, 95% confidence intervals (CI) and actual p‐values (with significance assumed at p < 0.05) will be reported as appropriate. Methods will include the Chi‐squared test or exact tests, as appropriate. We will aim to perform pre‐specified regression analyses for the Activity Survey relating risk factors (situation, patient and technical factors) and immediate complications, with 95% CI and p‐values, where possible, including factors associated with the outcomes. The analysis of vignette responses will describe within‐person variability, rather than inter‐individual variability, with pre‐agreed statistical significance testing.

SECURE will not involve any study intervention or any change to concomitant care; therefore, there is no direct risk of patient harm. All elements of the study have been discussed with the NHS Health Research Authority (HRA), which advised that the project is not considered as research, can be classified as a service evaluation and does not require ethical approval. No patient identifiable data will be collected; therefore, patient consent is not required.

In England and Wales, Confidential Advisory Group (CAG) approval is not required, as confirmed using their online checklist [29]. In Scotland, the Electronic Data Research and Innovation Service (eDRIS) team at Public Health Scotland has advised that Public Benefit and Privacy Panel for Health and Social Care (PBPP) permission is not required. In Northern Ireland, SECURE has been approved as a service evaluation by the Health and Social Care Privacy Advisory Committee, with Data Access Agreement (DAA) forms completed for every Health and Social Care Trust.

The project has been reviewed, approved and registered as a service evaluation with the Information Governance department and Caldicott Guardian at the Sponsor site, University Hospitals Bristol and Weston (UHBW), Bristol, UK. Site leads will be mandated to provide evidence of study registration with the Information Governance department at each site before commencing data collection.

Pooled, non‐identifiable results from SECURE will be disseminated as widely as possible in the UK and internationally, through relevant bodies and our collaborators (DAS, SIVA, TRIC, TERN, RCoA and RCEM) to maximise impact and accessibility.

We will publish in peer‐reviewed scientific journals, present at conferences, provide feedback to patients through the RCoA Patient, Carer and Public Involvement and Engagement Group and communicate key findings through multiple channels, such as professional newsletters, society websites and social media platforms.

## Conclusion

Despite decades of changing clinical practice, many of the techniques and some of the equipment used in RSI have a limited evidence base. Surveys suggest widespread variation in practice but are potentially limited by discrepancies between self‐reported and actual behaviour.

SECURE is a national, resident‐led, prospective service evaluation which will characterise contemporary RSI in adults across NHS hospitals in the UK in the settings of anaesthesia, ICM and EM. The outcomes of the project are expected to influence national and international practice through updates to terminology, clinical opinion, research priorities and recommendations.

## Supporting information


**Appendix S1.** Primary outcome measures of ‘RSI preparedness’ for the SECURE Site Survey.
**Appendix S2.** Cross‐speciality (anaesthesia, intensive care medicine and emergency medicine) UK workforce estimates.
**Appendix S3.** UK Rapid Sequence Intubation (RSI) estimates.
**Appendix S4.** SECURE (RAFT‐5) Investigation Group members.
