# Supplementary material for: Study protocol for the SECURE (RAFT‐5) study: Service Evaluation of in‐hospital Cross‐speciality UK adult Rapid sequence intubation Events
Source: Anaesth Rep. 2026 Jul 8;14(2):e70071. doi: 10.1002/anr3.70071 (PMC13346352; doi:10.1002/anr3.70071)
Supplement: Supplementary file 1 — Appendix S1. Primary outcome measures of ‘RSI preparedness’ for the SECURE Site Survey. Appendix S2. Cross‐speciality (anaesthesia, intensive care medicine and emergency medicine) UK workforce estimates. Appendix S3. UK Rapid Sequence Intubation (RSI) estimates. Appendix S4. SECURE (RAFT‐5) Investigation Group members. [file ANR3-14-e70071-s001.docx]

**Supplementary Appendix 1 – primary outcome measures of “RSI preparedness” for the SECURE Site Survey**

All the measures below are based on DAS and PUMA airway/intubation guidelines [1-5], as adapted by the SECURE Investigation Group for the Site Survey.

| **Measure** | **Guideline** | **Guideline statement** | **Source from Site Survey** |
| --- | --- | --- | --- |
| Pre-oxygenation (any oxygen delivery method) | DAS 2025 guideline  DAS 2018 guideline  PUMA 2025 guideline | All patients **should** be pre-oxygenated before induction of general anaesthesia.  In the absence of respiratory failure, preoxygenate using a tight-fitting facemask, with 10–15 litres min−1 100% oxygen for 3 min. We do not recommend preoxygenation with a ‘Hudson-type’ facemask… In hypoxaemic patients, CPAP and non-invasive ventilation (NIV) may be beneficial. High-flow nasal oxygenation (HFNO) at flows between 30–70 litres min−1 is an alternative  **Recommended** component: Optimal pre-oxygenation | Site Survey section 2.1 and 2.2:  Piped wall oxygen OR Oxygen cylinder  **AND**  Self-inflating bag-valve-mask OR Waters’/Mapleson C circuit  **AND**  Clear anaesthetic face masks (sizes 3, 4, 5) |
| Wide-bore wall-mounted suction | DAS 2025 guideline  PUMA 2025 guideline | RSI consideration: Suction - On and at hand  **Recommended** component: Suction on and under pillow | Site Survey section 2.1:  Wall-mounted suction OR portable suction  **AND**  Yankuer/rigid suction catheters |
| Standard monitoring | DAS 2025 guideline  DAS 2018 guideline  DAS 2025 guideline  DAS 2018 guideline  DAS/OAA 2015 guideline  PUMA 2022 guideline | Institutions **should** provide equipment that enables patient monitoring in accordance with current Association of Anaesthetists recommendations.  Standard monitoring **should** include oximetry, waveform capnography, blood pressure, heart rate, ECG  Institutions **should** provide equipment that enables patient monitoring in accordance with current Association of Anaesthetists recommendations.  Patients undergoing general anaesthesia require … waveform capnography…After induction of anaesthesia, uninterrupted waveform capnography is essential to demonstrate effectiveness of facemask ventilation, and is the most sensitive and specific monitor to exclude oesophageal intubation.  It is **mandatory** to use waveform capnography to confirm intubation.  A sustained capnographic trace is the most reliable method of confirming tracheal intubation.  Exhaled carbon dioxide monitoring … **should** be available and used for all episodes of airway management. | Site Survey section 2.5:  **ALL**  Non-invasive blood pressure monitoring  (NIBP) cable and cuffs, Pulse Oximetry  (SpO2), Cardiac monitoring (3-Lead ECG),  Monitor block/screen, quantitative waveform capnography (with appropriate tubing and connector) |
| Rapid dose of IV induction agent | DAS 2025 guideline  DAS 2018 guideline  PUMA 2025 guideline | Propofol is the most widely used induction agent, providing favourable conditions for airway management.  RSI consideration: rapid onset of anaesthesia - dosing according to physiology.  We **recommend** … intravenous induction  **Recommended** component: pre-determined dose of induction agent – given as rapid IV bolus | Site Survey section 2.10:  **At least one**  induction agent |
| Neuromuscular blocking agent | DAS 2025 guideline  DAS 2018 guideline  PUMA 2025 guideline  DAS/OAA 2015 guideline | Neuromuscular blocking agents **should** be routinely used to facilitate tracheal intubation.  RSI consideration: Rapid onset of neuromuscular block  We **recommend** … a rapid-onset neuromuscular blocking agent  **Recommended** component: rapid onset NMBA  The use of high-dose rocuronium (1.0–1.2 mg.kg−1) with sugammadex backup is a suitable alternative to suxamethonium | Site Survey section 2.10:  Rocuronium  **OR**  Suxamethonium |
| Emergency drugs | DAS 2025 guideline  DAS 2018 guideline | …useful for clinicians to prepare for haemodynamic instability regardless of induction agent.  Haemodynamic status **should** be optimised before induction of anaesthesia, and those at risk of ongoing haemodynamic instability **should** have a team member nominated to manage this.  A vasopressor or inotrope **should** be immediately available for bolus and infusion during induction and intubation. In shock states, a vasopressor **should** also be considered before induction. | Site Survey 2.10:  **At least one** **of**  Metaraminol  Noradrenaline  Phenylephrine  **AND**  Adrenaline 1mg (10000) OR adrenaline 1mg (1:1000)  **AND**  Atropine |
| Video-laryngoscopy (standard and hyperangulated blades) | DAS 2025 guideline  DAS 2018 guideline  PUMA 2025 guideline  DAS/OAA 2015 guideline | Videolaryngoscope **should be used first line** to facilitate tracheal intubation whenever possible.  RSI consideration: VL – with optimised ergonomics for shared view  *(Not included here as DAS 2025 guideline updates evidence based to recommend VL as 1^st^ line)*  **Recommended** component: video-laryngoscope  … it has been suggested that a videolaryngoscope should be the first-line device for all tracheal intubations.  A videolaryngoscope **should** be immediately available for all obstetric general anaesthetics. | Site Survey section 2.6:  Indirect: Videolaryngoscope with Mac 3 and 4  **AND**  Indirect: Videolaryngoscope with hyper-angulated blade |
| Tracheal introducers (bougie and stylet) | DAS 2025 guideline  DAS 2018 guideline  PUMA 2025 guideline  DAS/OAA 2015 guideline | RSI consideration: Tracheal tube introducers - Bougie or stylet prepared  Use of a bougie or stylet is recommended when the laryngeal opening is poorly seen (Grade 2b or 3a views) or when using a hyperangulated videolaryngoscope.  **Recommended** component: Tracheal introducer prepared  Insertion of the tracheal tube can be facilitated with the use of a tracheal tube introducer (bougie) or a stylet. | Site Survey section 2.6:  Standard bougie (non-dynamic)  **OR**  Rigid stylet (of same manufacturer as VL) |
| Range of ETT sizes | DAS 2018 guideline  DAS/OAA 2015 guideline | …the tracheal tube **should** be wide enough to enable suction catheter… during difficult airway management, a smaller (e.g. 6.0 mm inner diameter) or non-specialized tracheal tube may facilitate easier intubation.  Small tracheal tubes (e.g. size 7.0) **should** be used routinely to improve the success rate and minimise trauma. | Site Survey section 2.6:  ≥2 Tracheal tubes, cuffed, size range  6.0 - 8.0mm ID |
| 2^nd^ generation supra-glottic airway device  (DAS plan B) | DAS 2025 guideline  DAS 2018 guideline  DAS/OAA 2015 guideline | If tracheal intubation fails (Plan A), a second-generation supraglottic airway device **should** be used to attempt rescue.  During airway rescue, SGA insertion is initially preferable to attempted facemask ventilation because SGAs may frequently enable oxygenation.  Second-generation SGAs **should** be immediately available in all locations where intubation of critically ill patients is attempted.  A second-generation SAD with a gastric drain tube is recommended | Site Survey section 2.6:  2nd generation supraglottic airway devices (sizes 3, 4 and 5) |
| Facemask ventilation  (DAS Plan C) | DAS 2025 guideline  DAS 2018 guideline | Facemask ventilation **should** be used to maintain oxygenation between attempts at airway instrumentation. (PUMA Recommended: Apnoeic oxygenation between attempts at laryngoscopy)  When tracheal intubation and ventilation through a supraglottic airway device have failed, a final attempt at oxygenation by facemask ventilation should be made.  Following failed intubation attempts, experienced operators enter a phase of airway rescue, attempting SGA placement interspersed with attempted facemask ventilation. This is recognised by the ‘Vortex approach’, which we **recommend**. | Site Survey section 2.1 and 2.2:  Piped wall oxygen OR Oxygen cylinder  **AND**  Self-inflating bag-valve-mask OR Waters’/Mapleson C circuit  **AND**  Clear anaesthetic face masks (sizes 3, 4, 5) |
| Airway adjuncts (DAS plan C) | DAS 2025 guideline  DAS 2018 guideline  DAS/OAA 2015 guideline | For this final rescue attempt at facemask ventilation:  … c) use airway adjuncts  …oral airway adjuncts… may improve facemask ventilation.  An oropharyngeal airway… **should** be used if facemask ventilation is difficult | Site Survey section 2.1:  Oropharyngeal airways (sizes 2, 3, and 4) |
| eFONA (scalpel-bougie-tube technique)  (DAS plan D) | DAS 2025 guideline  DAS 2018 guideline | The **required** equipment for eFONA are: a scalpel with a number 10 blade, a bougie, and a size 6.0 tracheal tube  We **recommend** a scalpel-bougie-tube cricothyroidotomy technique…scalpel blade (size 10 or 20) …insertion of a bougie as a guide for a 5.0–6.0 mm tracheal tube. | Site Survey section 2.8:  Difficult airway trolley stocked as per DAS difficult airway principles ticked yes  **OR**  Under what items ‘NOT being included’ in Drawer D Plan D Emergency Front of Neck none ticked |

1. Ahmad I, El‐Boghdadly K, Iliff H, et al. Difficult Airway Society 2025 guidelines for management of unanticipated difficult tracheal intubation in adults. Br J Anaesth 2026; 136: 283–307.
2. Higgs A, McGrath B, Goddard C, et al. Guidelines for the management of tracheal intubation in critically ill adults. Br J of Anaesth 2018; 120 ; 323 – 352.
3. Mushambi, M, Kinsella SM, Popat M, et al. Obstetric Anaesthetists' Association and Difficult Airway Society guidelines for the management of difficult and failed tracheal intubation in obstetrics. 2015. Anaesthesia 2015; 70 ; 1286-1306.
4. Chrimes N. Preview content: universal guidelines for rapid sequence intubation. Project for the Universal Management of Airways (PUMA) Group. 2025. (accessed on 09/12/2025).
5. Chrimes N, Higgs A, Hagberg CA et al. Preventing unrecognised oesophageal intubation: a consensus guideline from the Project for Universal Management of Airways and international airway societies. Anaesthesia 2022 ; 77 ; 1395-1415.

**Supplementary Appendix 2.** Cross-specialty (anaesthesia, intensive care medicine and emergency medicine) UK workforce estimates.

Table 1. Practitioner numbers by training background and job role/grade.

| **Training background** | Consultants  *(n)* | *AS*  *(n)* | SAS  *(n)* | SG /  LEDs  *(n)* | Specialty registrars  *(n)* | Core trainees  *(n)* | Other*  *(n)* | **Totals**  *(n)* |
| --- | --- | --- | --- | --- | --- | --- | --- | --- |
| Anaesthesia only | 6548 | 417 | 1343 | 18 | 2743 | 2116 | 232 | 13,674 |
| Anaesthesia and ICM | 1861 | U/K | U/K | U/K | 489 | N/A | N/A | 2350 |
| ICM only | 209 | 21 | 58 | 22 | 522 | N/A | 210 | 1042 |
| ICM and EM | 31 | U/K | U/K | U/K | 129 | N/A | N/A | 160 |
| EM only | 2959 | 235 | 89 | 1434 | 2913 | 2514 | U/K | 10,144 |
| **Totals** | 11,608 | 673 | 1490 | 1474 | 6796 | 4630 | 442 | **27,370** |

AS, associate specialist; SAS, specialist and specialty doctors; SG, staff grade; LED, locally employed doctors; U/K, unknown; ICM, intensive care medicine; EM, emergency medicine.

*Includes qualified physician assistants in anaesthesia (or equivalent) and qualified advanced critical care practitioners (or equivalent).

**Practitioner numbers**

Anaesthesia practitioners, including practitioners in both anaesthesia and ICM.

NHS Hospital and Community Health Services (HCHS) Workforce Data: England [1]

- Consultants: 6,538
- Associate Specialists (AS): 417
- Specialty and Associate Specialist (SAS) doctors: 1251
- Staff Grades / Locally Employed Doctors (LEDs): 18
- Registrars: 2886
- Core Trainees: 2116

NHS Scotland Workforce Data (includes practitioners in both anaesthesia and ICM) [2]

- Consultants: 626
- Registrars / Core Trainees: 346
- Others: 92

NHS Wales and Northern Ireland Workforce Data – not publicly accessible

Anaesthesia and ICM practitioners

FICM Workforce Data [3-4] – totals are estimated because many ICM practitioners are dual-specialty:

- Consultants (approximately 2092 of whom about 89% practice dually with anaesthesia): 1861
- Registrars (approximately 1140 of whom about 43% practice dually with anaesthesia): 489
- Others: unknown

ICM only practitioners

FICM Workforce Data [3-5] – totals are estimated because many ICM practitioners are dual specialty:

- Consultants (approximately 2092 of whom around 10% do not dual practice with anaesthesia or EM): 209
- Qualified Advanced Critical Care Practitioners (or equivalent): 210
- Registrars (approximately 1140 of whom around 46% do not dual practice with anaesthesia or EM): 522
- Others: unknown

NHS Hospital and Community Health Services (HCHS) Workforce Data: England [1]

- Associate Specialists (AS): 21
- Specialty and Associate Specialist (SAS) doctors: 58
- Staff grades / Locally Employed Doctors (LEDs): 22
- Others: unknown

NHS Scotland, Wales and Northern Ireland Workforce Data – not publicly accessible

EM and ICM practitioners

FICM Census data [3]

- Consultants: 31
- Registrars: 129
- Others: unknown

EM practitioners, including in practitioners in both EM and ICM

NHS Hospital and Community Health Services (HCHS) Workforce Data: England [1]

- Consultants: 2880
- Associate Specialists (AS): 235
- Specialty and Associate Specialist (SAS) Doctors: 1434
- Staff Grades / Locally Employed Doctors (LEDs): 38
- Registrars: 2764
- Core trainees: 2514

NHS Scotland Workforce Data [2]

- Consultants: 110
- Staff Grades / Locally Employed Doctors (LEDs): 51
- Registrars / Core trainees: 278

NHS Wales and Northern Ireland Workforce Data – not publicly accessible

**References**

1. NHS England. NHS Hospital & Community Health Services (HCHS) monthly workforce statistics - Staff in NHS trusts and other core organisations, 2025. <https://digital.nhs.uk/data-and-information/publications/statistical/nhs-workforce-statistics/december-2025> (accessed 12/03/2026).
2. NHS Education for Scotland. NHS Scotland Workforce, 2025. <https://turasdata.nes.nhs.scot/data-and-reports/official-workforce-statistics/all-official-statistics-publications/03-march-2026-workforce> (accessed 12/03/2026).
3. Porter R, Williams M. Faculty of Intensive Care Medicine (FICM). FICM census, 2022. <https://www.ficm.ac.uk/careersworkforceworkforce/census> (accessed 12/03/2026).
4. Faculty of Intensive Care Medicine (FICM). FICM Training Quality Report, 2025. <https://ficm.ac.uk/sites/ficm/files/documents/2025-05/FICM%20Training%20Quality%20Report%202025.pdf> (accessed 12/03/2026).
5. Faculty of Intensive Care Medicine (FICM). Workforce Data Bank 2021, 2021. <https://www.ficm.ac.uk/sites/ficm/files/documents/2021-10/workforce_data_bank_2021_-_for_release.pdf> (accessed 12/03/2026).

**Supplementary Appendix 3.** UK Rapid Sequence Intubation (RSI) estimates.

All estimates are for acute hospital patients ≥16 years across the UK.

Total UK acute hospital beds (106,068 England; 10,330 Wales; 13,755 Scotland; 5186 Northern Ireland) is approximately 133,339 beds [1-4].

**Table 1.** Estimated in-hospital RSIs in adults ≥16 years per 14 days in the UK by different capture rates for SECURE.

| **Estimates by capture rate over 14 days for SECURE** | **Theatre RSIs**  **(n)** | **Out-of-theatre RSIs**  **(n)** | **All RSIs**  **(n)** |
| --- | --- | --- | --- |
| 100% | 5506 | 1895 | 7401 |
| 80% | 4405 | 1516 | 5921 |
| 60% | 3303 | 1137 | 4440 |
| 40% | 2202 | 758 | 2960 |
| 20% | 1101 | 379 | 1480 |
| 10% | 550 | 190 | 740 |

**Previous out-of-theatre cross-specialty RSIs studies.**

- Gibson et al. (2023) – 106 RSIs out-of-theatre (all areas and cross-specialty teams) over about 3850 acute hospital beds in 28 days (99% estimated capture rate) [5]. Rate of 0.014 RSIs per acute hospital bed per 14 days.
- Reid et al. (2006) - Intubations by critical care team in all out-of-theatre areas of Portsmouth Hospitals NHS Trust over 6 months (2000-2001): 199 RSIs in total for 1055 acute hospital bed [6,7]. Rate of 0.014 RSIs per acute hospital bed per 14 days.

**Previous emergency department-specific cross-specialty RSIs studies.**

NAP4 did not measure ED RSI numbers specifically [8]. NAP4 references a figure from Benger et al. 2010: “Approximately 1 in 800 patients attending the emergency department will undergo rapid sequence induction of anaesthesia and tracheal intubation (RSI), and this equates to approximately 20,000 patients every year in the UK” [9].

- Benger et al. (2010): 64 (26.8%) of EDs, 14 days, 199 RSIs ≥16 years (90% capture). In 2008, about 77% of all ED attendances (176,036) were ≥16 years (135,547) [9].

Rate of RSIs: 3.1 per 14 days, 1 in 680 ED attendances.

- Simpson et al. (2006): 5 years SGH Glasgow – 225,000 attendances and 255 RSIs in 5 years [10].

Rate of RSIs: 2.1 per 14 days, 1 in 880 ED attendances.

- Reid et al. (2006): 6 months in Portsmouth Hospitals NHS Trust (adults) – 37% of out-of-theatre RSIs (74 of 199 patients) were in ED versus 63% in other out-of-theatre hospital areas [6]. Separate estimate for this Trust of 56,500 ED attendances in 6 months [11].

Rate of RSIs: 6.0 per 14 days, 1 in 764 ED attendances.

- Stevenson et al. (2006): 2003-2006 (40 months), Crossfield Hospital in Scotland; 58,000 ED attendances per annum on average and 181 RSIs [12].

Rate of RSIs: 2.3 per 14 days, 1 in 1068 ED attendances.

Mean rate of RSIs across these four single-centre studies – 1 in 848 ED attendances (range 1 in 680 – 1068). Based on UK ED attendance NHS Digital data April 2020 – March 2025 (England and Wales ≥16 years; Scotland 75% patients ≥16 years; Northern Ireland 65% patients ≥16 years) [10, 13-20]. Mean number of 919 RSIs per 14 days (range 739 – 1029).

**Estimates based on previous out-of-theatre and ED-specific RSI studies**

Around 50% of out-of-theatre RSIs appear to be performed in the emergency department (ED) according to previous studies. The ED rate of RSIs was consistent across the studies, in keeping with the figure quoted in the NAP4 full report. Notably the rate of RSIs appeared to be identical in both the out-of-theatre studies described despite geographical and time separation of 17 years.

**Table 2.** Estimates of out-of-theatre RSIs (including ED subgroup) over 14 days in UK by different captures rates for SECURE.

| **Estimates by capture rate over 14 days for SECURE** | **Out-of-theatre RSIs**  **(2 studies)**  **(n)** | **Emergency department RSIs**  **(4 studies)**  **n (range)** |
| --- | --- | --- |
| **100%** | 1895 | 919 (739 - 1029) |
| **80%** | 1516 | 735 (591 - 823) |
| **60%** | 1137 | 551 (443 - 617) |
| **40%** | 758 | 368 (296 - 412) |
| **20%** | 379 | 184 (148 - 206) |
| **10%** | 190 | 92 (74 - 103) |

**Previous theatre RSIs studies (NAP5-7)**

Data on RSIs was estimated as part of the NAP5 study over a seven-day period. We have derived estimated RSI numbers for NAP5-7 over a 14-day period (assuming an identical RSI rate for NAP5-6) [21-23]. The RSI rate for all obstetric theatres is extrapolated from the NAP5 RSI rate reported in caesarean birth cases (92.2%) [21].

**Table 3.** Estimated theatre RSIs by sub-group over 14 days in the UK (with ED procedure/imaging RSIs subtracted from the non-obstetric theatre RSI numbers).

| **NAP study** | **Year data collected** | **Non-obstetric theatres**  **(n)** | **Obstetric**  **theatres (caesarean births)**  **(n)** | **All**  **theatre areas**  **(n)** |
| --- | --- | --- | --- | --- |
| **5** | 2013 | 5776 | 440 | 6216 |
| **6** | 2016 | 5026 | 382 | 5408 |
| **7** | 2021^*^ | 4654 | 241 | 4895 |
| **Mean** | N/A | 5152 | 354 | **5506** |

* RSI numbers likely affected by COVID-19 pandemic.

**Table 4.** Estimated NAP5-7 RSIs by anaesthetic team (all theatre areas) for acute UK hospitals.

| **Estimates by capture rate over 14 days for SECURE** | **All theatre RSIs**  **(n)** |
| --- | --- |
| **100%** | 5506 |
| **80%** | 4405 |
| **60%** | 3303 |
| **40%** | 2202 |
| **20%** | 1101 |
| **10%** | 550 |

**Full breakdown of derived NAP5 data: General anaesthetics with RSI by anaesthetic team**

267 centres with local coordinators in UK – activity survey over two days (September 2013)

NAP5 scaled two days up to days per annum, calculating with factor of 180.68x

14,790 GA out of 17,639 total cases (83.8%) over 2 days = 2,766,600 GA per annum

RSI numbers (based on NAP5 data below):

1. Theatre GA RSIs numbers (excluding caesarean births) = 3189 per week ≥16 years

NAP5 Activity Survey estimates:

Section 27.28 RSI in 7.4% of non-caesarean GA cases

Section 27.24 2,766,600 total GAs per annum

Section 15.23 488,500 GAs in patients aged <16 per annum

Section 16.40 8,000 CS GAs per annum

Section 17.15 29,000 ED and ICU GAs per annum

Figure 27.20 RSI in 7.4% of GAs ≥16 years

Calculation using NAP5 estimates:

Step 1. (2,766,600 – 488,500) = 2,278,100 GAs ≥16 years per annum

Step 2. (2,278,100 – 8000 – 29,000) = 2,241,100 non-caesarean GAs ≥16 years per annum

Step 3. (2,241,100 / 52) = 43,098 non-caesarean theatre GAs ≥16 years per week

Step 4. (43,656 * 7.4%) = 3189 non-caesarean theatre GAs with RSI ≥16 years per week

Result: Theatre RSIs numbers (excluding caesarean births) ≥16 years = 3189 per week

1. GA caesarean births with RSI = 220 per week ≥16 years

NAP5 Activity Survey estimates:

Section 27.16 “Obstetric cases accounted for 8.9% of all activity (326,500 per year) of which only 10% involved GA.”

Section 27.16 noted but NAP5 Obstetric Chapter 16 has a different, detailed breakdown:

Section 27.28 RSI in 92.2% of caesarean birth GAs

Figure 16.2 226,000 analysable out of 352,000 obstetric cases (scaling factor x1.55)

Figure 16.2 95,000 caesarean births out of 226,000 analysable obstetric cases (42.0%)

Figure 16.2 8000 GA caesarean births out of 95,000 caesarean births per annum (8.4%)

Calculation using NAP5 estimates:

Step 1. (1.55 * 8000) = 12,460 GA caesarean births per annum

Step 2. (12,460 / 52) = 239 GA caesarean births per week

Step 3. (239 * 92.2%) = 220 GA caesarean births with RSI per week

Result: GA caesarean births with RSI = 220 per week

1. ED and ICU RSIs = 301 per week, including children

NAP5 Activity Survey inclusion criteria for ED/ICU RSIs by anaesthetists:

27.10 It included patients on the ICU in whom unconsciousness was induced or maintained for any surgical procedure, whether in theatre or at the bedside or for a diagnostic or radiological procedure, but it did not include ICU management with sedation. It also included emergency department (ED) cases, such as cases of trauma, where an anaesthetist secured the airway and transferred the patient to a site of a procedure.

NAP5 Activity Survey estimates:

17.15 The NAP5 Activity Survey provides an estimates of 29,000 general anaesthetics per year administered by anaesthetists in either the ICU or the ED (equivalent to approximately 1% of all UK anaesthetist-delivered general anaesthetics), and 54% involved RSI.

Calculation using NAP5 estimates:

Step 1. 29,000 / 52 = 557 GAs in ED or ICU per week

Step 2. (557 * 54%) = 301 GAs with RSI in ED or ICU per week

Result: ED and ICU RSIs = 301 per week, including children

NB: specific data for patients ≥ 16 years not available in NAP5 report

**Full breakdown of derived NAP6 data: GAs with RSIs by anaesthetic team**

NAP6 scaled 2 days (October 2016) up to numbers per annum

Total cases 3,126,067 per annum – 2,394,847 GAs per annum

RSI numbers (scaled from NAP5 data*):

1. Theatre GA RSIs numbers, excluding caesarean births = 2774 per week ≥16 years
2. GA CS with RSI = 191 per week ≥16 years
3. ED and ICU RSIs = 261 per week, including children

* NAP5 data:

GAs in ≥16 years (2,766,600 - 488,500) = 2,288,100 (82.7% ≥16 years)

2,251,100 GAs (excluding caesarean births and ED/ICU) ≥16 years (81.4% of GAs) - 7.4% RSI rate

12,460 GA caesarean births (scaled up 1.55x) out of 2,760,600 total GAs (0.45%) - 92.2% RSI rate

29,000 GAs in ED/ICU of 2,760,600 GAs (1.05%) - 54% RSI rate

**Full breakdown of derived NAP7 data: GAs with RSIs by anaesthetic team**

NB: numbers during NAP7 likely affected by COVID-19 pandemic

NAP7 scaled 4 days (November 2021) up to numbers per annum

16,604 GAs out of 20,288 total cases (81.8%)

Total cases 2,710,618 per annum – 2,217,285 GAs per annum

RSI numbers (scaled from NAP5 data*):

1. Theatre GA RSIs numbers, excluding caesarean births = 2568 per week ≥16 years
2. GA caesarean births with RSI = 176 per week ≥16 years
3. ED and ICU RSIs = 241 per week, including children

* NAP5 data:

GAs in ≥16 years (2,766,600 - 488,500) = 2,288,100 (82.7% ≥16 years)

2,251,100 GAs (excluding caesarean births and ED/ICU) ≥16 years (81.4% of GAs) - 7.4% RSI rate

12,460 GA caesarean births (scaled up 1.55x) out of 2,760,600 total GAs (0.45%) - 92.2% RSI rate

29,000 GAs in ED/ICU of 2,760,600 GAs (1.05%) - 54% RSI rate

**References**

1. NHS England. Bed Availability and Occupancy Data – Overnight, Quarter 4 2024-25, 2025. [www.england.nhs.uk/statistics/statistical-work-areas/bed-availability-and-occupancy/bed-data-overnight/](https://www.england.nhs.uk/statistics/statistical-work-areas/bed-availability-and-occupancy/bed-data-overnight/) (accessed 23/06/2025).
2. StatsWales. Monthly NHS beds data by measure, site and specialty: March 2014 onwards, 2025. [www.statswales.gov.wales/Catalogue/Health-and-Social-Care/NHS-Hospital-Activity/NHS-Beds/nhsbeds-by-organisation-specialty-month](https://www.statswales.gov.wales/Catalogue/Health-and-Social-Care/NHS-Hospital-Activity/NHS-Beds/nhsbeds-by-organisation-specialty-month) (accessed 23/06/2025).
3. Public Health Scotland. Acute hospital activity and NHS beds information (annual), 2024. [www.publichealthscotland.scot/media/29229/2024-09-24-annual-acuteactivity-report.pdf](https://www.publichealthscotland.scot/media/29229/2024-09-24-annual-acuteactivity-report.pdf) (accessed 23/06/2025).
4. Department of Health. Northern Ireland Inpatient Activity Statistics 2023/24, 2024. [www.datavis.nisra.gov.uk/health/ni-inpatient-stats-23-24.html](https://www.datavis.nisra.gov.uk/health/ni-inpatient-stats-23-24.html) (accessed 23/06/2025).
5. Gibson J, Leckie T, Hayward J, et al. Non-theatre emergency airway management: a multicentre prospective observational study. Anaesthesia 2023; 78 ; 1338-46.
6. Reid C, Chan L, Tweeddale M. The who, where, and what of rapid sequence intubation: prospective observational study of emergency RSI outside the operating theatre. Em Med J 2006 ; 21 ; 296-301.
7. NHS England. Acute hospital bed data for Portsmouth Hospitals NHS Trust: Bed Availability and Occupancy Data – Overnight 2006-2007, 2013. [www.england.nhs.uk/statistics/wp-content/uploads/sites/2/2013/04/Download-%E2%80%93-NHS-Organisations-in-England-2006-07-XLS-399K.xls](https://www.england.nhs.uk/statistics/wp-content/uploads/sites/2/2013/04/Download-%E2%80%93-NHS-Organisations-in-England-2006-07-XLS-399K.xls) (accessed 23/06/2025).
8. Cook TM, Woodall N, Frerk C, et al. Fourth National Audit Project of the Royal College of Anaesthetists and Difficult Airway Society. Major complications of airway management in the United Kingdom. Report and findings. London: Royal College of Anaesthetists, 2011.
9. Benger J and Hopkinson S. Rapid sequence induction of anaesthesia in UK emergency departments: a national census. *Em Med J* 2011; 28 ; 217-20.
10. Simpson G, Munro P, Graham C. Rapid sequence intubation in the emergency department: 5 year trends. Em Med J 2006; 23 ; 54-6.
11. Department of Health. ED attendance data for Portsmouth Hospitals NHS Trust: Archive - A&E Attendances Annual figures 2001-02, 2008. <https://webarchive.nationalarchives.gov.uk/ukgwa/20130105020048/http://www.dh.gov.uk/en/Publicationsandstatistics/Statistics/Performancedataandstatistics/AccidentandEmergency/DH_087973> (accessed 23/06/2025).
12. Stevenson V, Graham C, Hall R, et al. Tracheal intubation in the emergency department: the Scottish district hospital perspective. *Em Med J* 2007; 24 ; 394-7.
13. NHS Digital, NHS England. Hospital Accident and Emergency Activity 2021-22: Table 6, 2022. [www.digital.nhs.uk/data-and-information/publications/statistical/hospital-accident--emergency-activity/2021-22](https://www.digital.nhs.uk/data-and-information/publications/statistical/hospital-accident--emergency-activity/2021-22) (accessed 22/06/2025).
14. NHS Digital, NHS England. Hospital Accident and Emergency Activity: National Report Tables 2023-24, 2024. [www.digital.nhs.uk/data-and-information/publications/statistical/hospital-accident--emergency-activity/2023-24](https://www.digital.nhs.uk/data-and-information/publications/statistical/hospital-accident--emergency-activity/2023-24) (accessed 22/06/2025).
15. StatsWales. Number of attendances in NHS Wales emergency departments by age band, sex and site 2022-2024, 2024. [www.statswales.gov.wales/Catalogue/Health-and-Social-Care/NHS-Hospital-Waiting-Times/emergency-department/emergencyattendances-by-age-sex-site](https://www.statswales.gov.wales/Catalogue/Health-and-Social-Care/NHS-Hospital-Waiting-Times/emergency-department/emergencyattendances-by-age-sex-site) (accessed 23/06/2025).
16. Public Health Scotland. Accident and emergency: Downloads and open data, 2025. [www.publichealthscotland.scot/healthcare-system/urgent-and-unscheduled-care/accident-and-emergency/downloads-and-open-data/our-downloads/](https://www.publichealthscotland.scot/healthcare-system/urgent-and-unscheduled-care/accident-and-emergency/downloads-and-open-data/our-downloads/) (accessed 23/06/2025).
17. Public Health Scotland. Accident and emergency: Interactive charts, who attends, 2025. [www.publichealthscotland.scot/healthcare-system/urgent-and-unscheduled-care/accident-and-emergency/interactive-charts/who-attends/](https://www.publichealthscotland.scot/healthcare-system/urgent-and-unscheduled-care/accident-and-emergency/interactive-charts/who-attends/) (accessed 24/06/2025).
18. Department of Health Northern Ireland. Hospital statistics: emergency care activity 2023/24, 2024. [www.health-ni.gov.uk/publications/hospital-statistics-emergency-care-activity-202324](https://www.health-ni.gov.uk/publications/hospital-statistics-emergency-care-activity-202324) (accessed 23/06/2025).
19. Department of Health Northern Ireland. Hospital statistics: emergency care activity 2022/23, 2023. [www.health-ni.gov.uk/sites/default/files/publications/health/hs-emergency-care-22-23.pdf](https://www.health-ni.gov.uk/sites/default/files/publications/health/hs-emergency-care-22-23.pdf) (accessed 23/06/2025).
20. Pandit JJ, Cook TM et al. Fifth National Audit Project of the Royal College of Anaesthetists and the Association of Anaesthetists of Great Britain and Ireland. Accidental Awareness during General Anaesthesia in the United Kingdom and Ireland. Report and Findings. London: Royal College of Anaesthetists. 2014.
21. Cook TM, Harper NJN et al. Sixth National Audit Project of the Royal College of Anaesthetists. Perioperative Anaphylaxis. Report and Findings. London: Royal College of Anaesthetists. 2018.
22. Cook TM, Oglesby F, Kane AD, et al. Airway and respiratory complications: NAP7 report. Anaesthesia 2024; 79: 368–379.

**Supplementary Appendix 4.** SECURE (RAFT-5) Investigation Group members

Resident research networks/groups:

- RAFT (Research and Audit Federation of anaesthetists in Training)
- STAR (Severn Trainee Anaesthetic Research group)
  - official lead Resident Research Network (RRN) for RAFT-5
- TERN (Trainee Emergency Research Network)
- TRIC (Trainee Research in Intensive Care)
- SEARCH (South East Anaesthetic Research Chain)

Steering Committee members:

- Chief Investigator (CI): Jonathan Barnes (Consultant Anaesthetist)
- SECURE RAFT Chair: Thomas Davies (RAFT)
- SECURE STAR Chairs: Thomas Baumer (STAR) and Swati Gupta (STAR)
- Sara Tomassini (RAFT)
- Amelia Van Manen (RAFT)
- Suzanne Harrogate (STAR)
- Aravind Ramesh (STAR)
- David Ritchie (RAFT)

Consultant expert advisers:

- Professor Tim Cook – Anaesthetic and Intensive Care Consultant, Royal United Hospitals Bath NHS FT; Honorary Professor of Anaesthesia at University of Bristol
- Dr Sandeep Sudan – Anaesthetic Consultant, University Hospital Sussex NHS FT; RCoA Airway Lead; DAS Committee Member
- Professor Jonathan Benger – ED Consultant, University Hospitals Bristol and Weston NHS FT – Bristol NHS Group; Professor of Emergency Care at University of West England
- Dr Irene Grossi – Emergency medicine consultant, UHBW NHS FT – Bristol NHS Group; EMAR (Emergency Medicine Airway Registry) Lead

STAR group committee – doctors-in-training:

- Thomas Baumer (STAR Executive Chair)
- Swati Gupta (STAR Incoming Executive Chair)
- Aravind Ramesh (STAR Secretary)
- Suzanne Harrogate (STAR Anaesthetic Chair)
- Thomas Cloke (STAR ICM Chair)
- Carys Lim (STAR Treasurer)
- Jessica Casey (STAR Membership Secretary)
- Jessica Henry (STAR Liaison Lead)
- Maeve McLaughlin (STAR Liaison Lead)
- Oliver Barker (STAR Events Lead)
- Callum Taylor (STAR Website/IT Lead)

RAFT national committee – doctors-in-training:

- Thomas Davies (RAFT Chair)
- Benjamin Milne (RAFT ICM Vice-chair)
- John O'Rourke (RAFT Anaesthetic Vice-chair)
- David Ritchie (RAFT Treasurer)
- Inez Armstrong (RAFT Events Lead)
- Sara Tomassini  (RAFT Secretary)
- Amelia Van Manen (RAFT IT/Education Lead)

Others – doctors-in-training:

- Luke Flower (TRIC Co-chair)
- Adam Boulton (TRIC Co-chair)
- Benjamin Clarke (TERN Chair)
- Lauren Bose (EMAR Collaborator)
- Roshan Ramasamy (SEARCH)
- Todd Leckie (SEARCH)
